# A modification of the Nikaidoh procedure in double-switch operation for congenitally corrected transposition of the great arteries

**DOI:** 10.1016/j.xjtc.2025.08.007

**Published:** 2025-08-28

**Authors:** Takahiro Ito, Mitsuru Aoki, Hiroshi Koshiyama, Ikuo Hagino

**Affiliations:** a Department of Cardiovascular Surgery, Chiba Children's Hospital, Chiba, Japan; b Department of Cardiovascular Surgery, Heart Institute of Japan, Tokyo Women's Medical University, Tokyo, Japan

**Figure:**
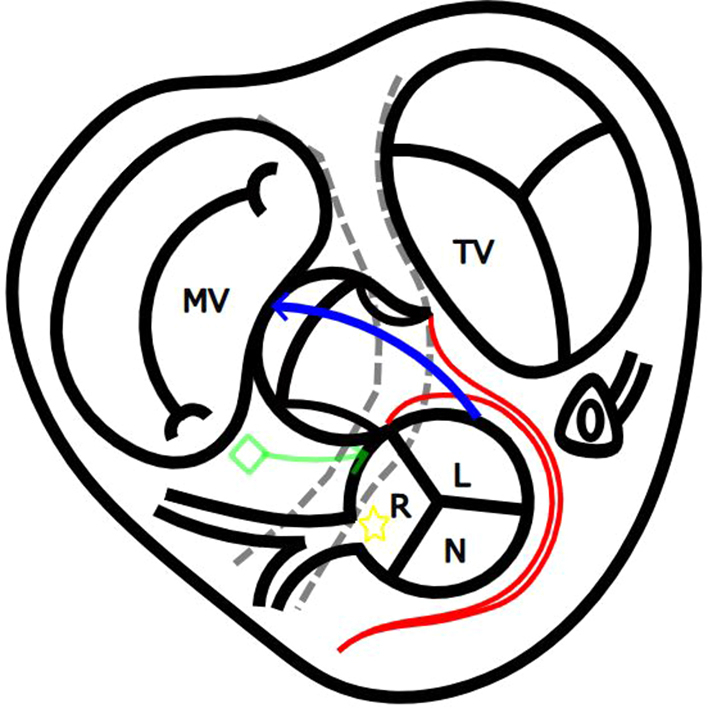
The aortic valve was rotated 120° counterclockwise and implanted in the PA valve annulus.

**Central Message**

Double-switch operation using a modification of Nikaidoh procedure is a good option for ccTGA with LVOTS when the VSD anatomy is unfavorable for intraventricular rerouting.

A 5-year-old girl underwent pulmonary artery banding at 11 months of age after a diagnosis of congenitally corrected transposition of the great arteries (ccTGA), ventricular septal defect (VSD), and mild pulmonary valve stenosis. The coronary arteries were of a normal mirror- image configuration. The pulmonary valve had a normal diameter but was bicuspid and thickened, making it unsuitable for an arterial switch operation.

An electrophysiology study was performed to assess the His bundle electrogram (HBE). A 4-Fr catheter with 10 electrodes was positioned to record HBE and a 4-Fr catheter with 8 electrodes was placed in the right atrium. HBE was carefully searched and recorded around the mitral annulus; the anterior atrioventricular (A-V) node was identified but a posterior A-V node was observed. The subaortic conal muscle was hypoplastic, and the VSD was of a subarterial type, although it was very shallow. This raised concerns about left ventricular outflow tract stenosis (LVOTS) after a Rastelli procedure. Therefore, aortic translocation was considered as the choice of procedure. Because of the hypoplastic conal muscle, a risk of anterior A-V node injury and resulting A-V block seemed high at the truncal block harvest. Therefore, we performed a modification of the Nikaidoh procedure, in which the aortic valve is rotated onto the left ventricular outflow tract around the anterior A-V node under the anterior coronary artery takeoff as a pivot. The operation was performed via a median sternotomy and with the patient under cardiopulmonary bypass.

The posteriorly located pulmonary artery (PA) was dissected and transected at the band. The PA valve was thickened and bicuspid with anterior-posterior commissures. Senning procedure was performed under induced cold ventricular fibrillation. The anterior right ventricular (RV) outflow was entered 15 mm below the aortic valve and extended toward the right-side A-V groove more away (25 mm) from the aortic valve (Video 1). The RV incision was extended leftwards approaching to the aortic valve (Figure 1, *A*).

**Figure 1:**
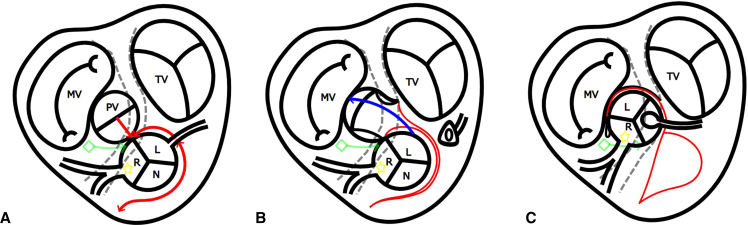
A modification of Nikaidoh procedure. A, The anterior RV incision was made away from the aortic valve and the posterior RV incision was made closer to the aortic valve. The posterior RV incision was extended into a junction of pulmonary and aortic annulus. The main pulmonary artery trunk was incised vertically at the annular incision. B, The posterior coronary artery was detached with a cuff of the Valsalva, the defect in the aortic Valsalva sinus was supplemented with a piece of the autologous pulmonary artery flap. The aortic valve was rotated 120° counterclockwise and implanted in the PA valve annulus. C, The posterior coronary artery was implanted in the aortic root, the previous noncoronary sinus. The RV-PA continuity was established with an expanded polytetrafluoroethylene tricuspid-valved conduit. *MV*, Mitral valve; *PV*, pulmonary valve; *TV*, tricuspid valve; ◇, atrioventricular node; ☆, pivot of aortic valve rotation; *R*, right coronary cusp; *L*, left coronary sinus; *N*, noncoronary sinus; *RV*, right ventricle; *PA*, pulmonary artery.

After the administration of cardioplegia, the ascending aorta was transected 10 mm above the sinotubular junction. The posterior coronary artery was detached with a cuff of the aortic Valsalva sinus, and the defect in the aortic Valsalva sinus was supplemented with an autologous PA patch. The left-side RV incision was extended to the aortic and pulmonary annular junction, merging with another vertical incision in the pulmonary trunk to open the PA annulus. The aortic valve was rotated 120° counterclockwise around the intact anterior ventricular septal junction, where anterior A-V node and the anterior coronary artery orifice were located nearby, as the pivot, and implanted in the PA valve annulus. The anterior pulmonary valve annulus just above the A-V conduction was closed by suturing the PA wall and valve remnants (Figure 1, *B* and *C*). The posterior coronary artery was implanted into the aortic root at the original non-facing sinus using the trap door technique. The ascending aorta was reconstructed by an end-to-end anastomosis. The VSD was closed with an expanded polytetrafluoroethylene patch. As a result of the aortic root rotation, a wide RV outflow opening was made at the left superior aspect of RV free wall. The RV-PA continuity was established by an expanded polytetrafluoroethylene tricuspid-valved conduit. A piece of free autologous PA strip was used to reinforce the left upper corner of the RV opening. The patient was weaned from cardiopulmonary bypass in sinus rhythm. Transesophageal echocardiography showed no aortic regurgitation, no mitral regurgitation, unobstructed venous channels, and good biventricular function. The patient was discharged 36 days after surgery. Postoperative catheterization and computed tomography demonstrated a wide straight left ventricular outflow tract and the RV-PA conduit position at the left of the sternum without compression (Figure 2).

**Figure 2:**
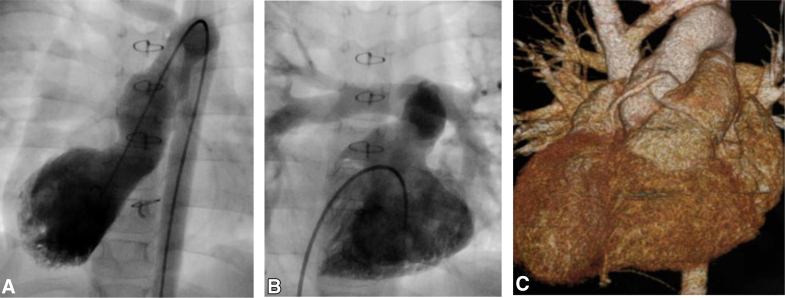
Postoperative catheterization and computed tomography scanning. A, Left ventricle angiography showed that the left ventricle outflow tract was straight. B and C, Right ventricle angiography and computed tomography showed that the conduit was on the left of the sternum, avoiding sternal compression.

This study was approved on October 23, 2023 (number 2023-0350). Written informed consent for the publication of her case details and images was obtained from the patient's parents.

## Discussion

Aortic translocation procedures, such as the Nikaidoh procedure and the half-turned truncal switch operation, are effective surgical options for TGA with LVOTS^1^^,^^2^; however, in ccTGA they carry a high risk of A-V block because of its specific characteristics in A-V conduction. The incidence of A-V block has been reported to be very high, ranging from 12% to 33%,^3^^,^^4^ when the aortic root is resected and implanted en block. Feins and colleagues^5^ have described the utility of intraoperative conduction mapping. Although it is technically feasible to confirm the location of the His bundle using a 3-dimensional map merged with 3-dimensional computed tomography images, this approach is not routinely used. HBE was defined by a discrete deflection between the atrial and ventricular electrograms 35 to 55 milliseconds before QRS onset. Furthermore, pacing is conducted from multiple sites within the right atrium to evaluate the presence of twin AV node. When the His bundle courses in close proximity of the pulmonary valve annulus, performing aortic translocation becomes challenging. In cases of severe pulmonary stenosis and relatively good atrial and ventricular septum alignment, a posterior bundle may also be present, forming a sling with the anterior bundle. In this case, the pulmonary valve annulus was normal in size, and the posterior A-V node was not demonstrated by electrophysiology study. The our modification of the Nikaidoh procedure, in which the aortic valve is rotated 120° counterclockwise around the anterior A-V node, can safely avoid A-V block. The unique feature of this procedure was the anterior oblique RV incision, which was away from the aortic valve and anterior A-V conduction, keeping their structural integrity, and leaving a wide opening for RV outflow reconstruction by a valved conduit without sternal compression. Use of the pulmonary valve and PA wall remnants further ensures protection of the A-V node and the mitral valve functions.

An aortic valve of ccTGA is not supported by the central fibrous skeleton, and there is concern about aortic root enlargement in the long term. In double-root translocation and the truncal switch operation, excision of the pulmonary annulus from the central fibrous skeleton might reduce the postoperative support of mitral and translocated aortic valves by the fibrous skeleton, and there is an increased concern of postoperative mitral and aortic regurgitation compared with the original Nikaidoh procedure. In our modification of the Nikaidoh procedure, the translocated aortic valve is supported posteriorly by the fibrous skeleton and anteriorly by the RV wall muscle with intact coronary blood supply, which may prevent aortic root enlargement in the remote stage.

## Conclusions

A double-switch operation using a modification of the Nikaidoh procedure is a good option for ccTGA with LVOTS when the VSD anatomy is unfavorable for intraventricular rerouting. The presented the modification of the Nikaidoh procedure is useful for avoiding A-V block.

## Conflict of Interest Statement

The authors reported no conflicts of interest.

The *Journal* policy requires editors and reviewers to disclose conflicts of interest and to decline handling or reviewing manuscripts for which they may have a conflict of interest. The editors and reviewers of this article have no conflicts of interest.

## Supplementary Data

Video 1 A modification of the Nikaidoh procedure in double-switch operation to avoid atrioventricular block in a 5-year-old girl with congenitally corrected transposition of the great arteries {S, L, L}. Video available at: https://www.jtcvs.org/article/S2666-2507(25)00346-3/fulltext.
