# Supplementary figures and images for: A modification of the Nikaidoh procedure in double-switch operation for congenitally corrected transposition of the great arteries
Source: JTCVS Tech. 2025 Aug 28;34:145–7. doi: 10.1016/j.xjtc.2025.08.007 (PMC12682962; doi:10.1016/j.xjtc.2025.08.007)

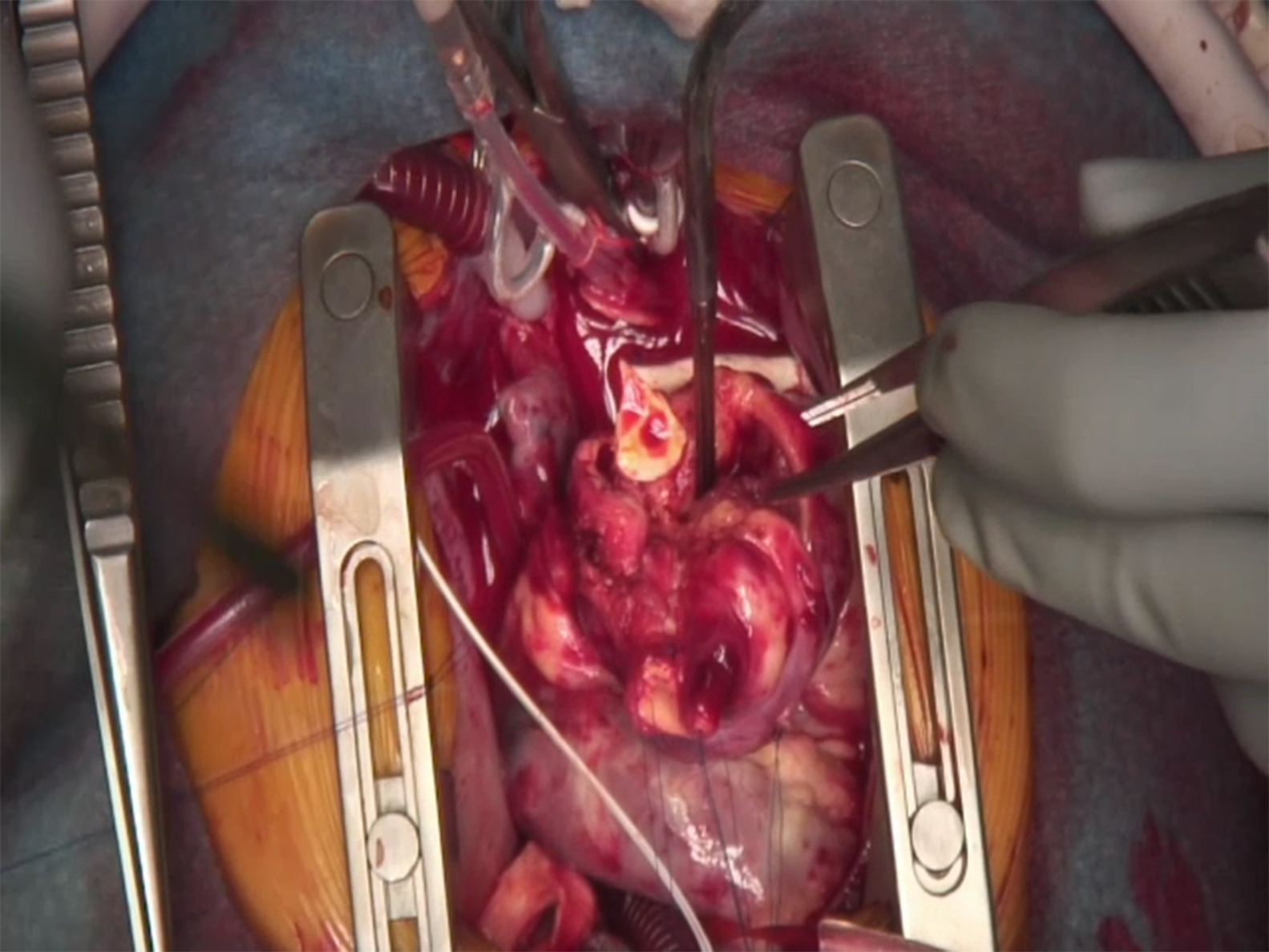

Supplement: Video 1 — A modification of the Nikaidoh procedure in double-switch operation to avoid atrioventricular block in a 5-year-old girl with congenitally corrected transposition of the great arteries {S, L, L}. Video available at: https://www.jtcvs.org/article/S2666-2507(25)00346-3/fulltext. [file fx2.jpg]
